# Evaluating additive versus interactive effects of copper and cadmium on *Daphnia pulex* life history

**DOI:** 10.1007/s11356-019-06622-9

**Published:** 2019-11-25

**Authors:** Shlair A. Sadeq, Andrew P. Beckerman

**Affiliations:** Department of Animal and Plant Sciences, University of Sheffield, Alfred Denny Building, Western Bank, Sheffield, S10 2TN UK

**Keywords:** Copper, Cadmium, *Daphnia pulex*, Genotypes, Life history, Response surface models

## Abstract

A key challenge of standard ecotoxicological risk assessment is to predict the sub-lethal risk of multiple contaminants on aquatic organisms. Our study assessed the sub-lethal mixture toxicity of copper (Cu) and cadmium (Cd) on *Daphnia pulex* and included manipulations of food level and assessment of three genotypes. We investigated the interaction between essential (Cu) and non-essential (Cd) metals on ingestion rate, reproduction, maturation time, size at maturity and somatic growth rate of three *D. pulex* genotypes, over 21 days and under standard and high food conditions. We explored the potential interaction of the metals on ingestion and life history by implementing a response surface experimental design combining control and two levels of Cu and Cd and their combinations. Overall, both metals reduced ingestion rates, reduced reproduction, delayed maturation, reduced body size at maturity and lowered somatic growth rate. Our results further indicated pervasive interactions between the metals; numerous instances where the effects of each metal were non-linear; the effect of a metal varied by *D. pulex* food levels (ingestion rate and size at maturity), and the effect of a metal varied by genotypes (reproduction). Apart from the maturation time and somatic growth rate, our results suggest that life history traits are affected in non-additive ways by three factors that are often discussed and rarely estimated together: mixtures of metals, genotypes and resource levels. Our data that are derived from exposing daphnids to two metals highlight how metals interact with each other and the context of food resource and genetic variation. While interactions make it harder to generate predictions, and ultimately water quality regulations about the effects of metals, those detected in this study appear to be tractable.

## Introduction

Metal toxicity is a worldwide concern arising from natural and anthropogenic discharges such as domestic effluents, agricultural and industrial activities (Ferreira et al. 2008; Shaw et al. 2017). At low and threshold concentrations, metals are known to induce a range of effects on living organisms and their communities, ranging from reductions in reproduction and growth to associated changes in food web interactions. Their persistence, their non-biodegradability and these associated effects on life history and species interactions ultimately impact on ecosystem services and human health (Monserrat et al. 2007; Nzengue et al. 2011; Piscia et al. 2015).

How stressors such as metals interact with each other and the environment remains a central question in ecology and ecotoxicology because most investigations have historically been limited to single stressors. However, numerous approaches now exist to assess whether the effects of multiple stressors are additive, synergistic or antagonistic, including methods linked to rejecting the null additive models of independent action or concentration addition through response surface experiments designed to detect species traits whether and how interactions among stressors manifest (Barata et al. 2006; Ferreira et al. 2008; Laskowski et al. 2010; Pavlaki et al. 2011).

Here, we performed a response surface experiment and evaluated the effects of Cu and Cd on feeding rates and four life history traits using *D. pulex* as a model organism. In aquatic communities, Cu and Cd are two major stressors among inorganic pollutants and have received much attention in risk assessment regulations (Bellavere and Gorbi 1981; Shuhaimi-Othman et al. 2010; Fernández-Gonzáles et al. 2011). Each metal has a different cellular and potentially ecological mode of action (e.g. impact on life history) because of the differences in each metal’s absorbability, solubility, chemical reactivity, transport and formation of complexes within the body (Shanker 2008, Stohs and Bagchi 1995).

At the molecular level, metal–metal interaction is a chemical reaction which can change the oxidation state of the metals, cleave organic radicals and change the state of organometallic compounds (Magos and Webb 1978). In organism’s bodies, metal–metal interactions (among essential and non-essential metals) may occur due to the similarity in physical and chemical properties among elements via the mechanisms of ionic and molecular mimicry (Brzóska and Moniuszko-Jakoniuk 2001; Bridges and Zalups 2005). These interactions may cause substantial alterations in the apparent properties of components as well as produce complexes that induce negative effects in organisms (Altenburger et al. 2013). For example, Cd effects arise from interactions with micro- and macro-elements (essential analogues) such as Ca, Zn, Cu and Se through ionic mimicry (Feng et al. 2018). Via ionic mimicry, species of a certain metal are able to mimic either the essential element or the cationic form of the element (Zalups and Ahmad 2003). Cadmium can act as an ionic mimic via substitution for other metal ions (mainly Zn^2+^, Cu^2+^ and Ca^2+^) in metalloenzymes (Brzóska and Moniuszko-Jakoniuk 2001). In contrast, molecular mimicry affects the binding of nucleophilic groups of certain biomolecules to metal ions (Zalups 2000). For example, Cd tends to bind to structures containing −SH groups, such as enzymes, proteins and nucleic acids (Stohs and Bagchi 1995).

Despite these emerging insights that offer cellular level hypotheses about how interactions (synergy or antagonism) between metals might arise, we still lack robust data on the outcomes of these cellular level effects, including how Cu and Cd combine to affect foraging and life history. Here, we focus on identifying whether the effects of Cu and Cd are additive or interactive on foraging and life history traits using three genotypes of *Daphnia pulex* exposed to Cu and Cd via a response surface experiment. We further replicated our experiment under standard and high food levels.

*Daphnia* spp. are a model system for detecting the effects of metal pollution in aquatic environments. They have a significant role in the aquatic food chain linking between algae, higher invertebrates and fish. Given their susceptibility to various contaminants, short generation times, easy culturing and high fecundity rates, they are widely used in experiments (Sarma and Nandini 2006; Colbourne et al. 2011). Furthermore, water flea individuals are clonal, reproducing asexually and populations are typically comprised of many genetically distinct genotypes that can respond differently to environmental pollutants. This makes it possible to explore easily how interactions among stressors may vary among genotypes of *Daphnia* spp. Several studies have identified substantial genetic variation in response to metals (Baird et al. 1990, 1991; Soares et al. 1992; Barata et al. 1998). Understanding whether there is a genetic variation in response to stressors is crucial to the management of natural water bodies which will typically be populated by multiple genotypes and eco-toxicological test outcomes which often use a single genotype.

Our work here builds on a rich body of literature reporting on the toxicity of each of Cu or Cd (Agra et al. 2011; Piscia et al. 2015; Gama-Flores et al. 2006; Guan and Wang 2006; Sadeq and Beckerman 2019). While there is no consistent guideline for the assessment of mixture toxicity, one current approach is to explore how the individual components combine in mixture using the concentration addition (CA) versus independent action (IA) models (Cedergreen et al. 2008). The CA model was historically formulated for exploring how chemicals with similar modes of action combine and assumes that compounds with similar modes of action will behave as if they are simply higher doses of a single compound. Critically, the dose of each compound is expected to combine additively on the response variables. In contrast, the IA model was developed against an assumption that the effects of the compounds, not the compounds themselves, behave additively and typically applies to chemicals with vastly different modes of action, for example, anthropogenic stress versus natural stress (Rodea-Palomares et al. 2015**)**.

Here, based on detail presented above, we assume that Cu and Cd operate with different modes of action and that effects may combine additively or with interaction (e.g. the IA assumptions). To assess this, we employ a response surface experiment and analysis, manipulating exposure to single and combined concentrations of each metal. We tested whether mixture concentrations of Cu and Cd affect ingestion rate, size at maturity, age at maturity, somatic growth rates and reproduction in an additive versus synergistic manner as revealed by patterns in a response surface, where the life history traits are measured along single and combined concentration gradients of Cu and Cd.

A long history of statistical inference from response surface theory forms the basis by which we draw conclusions: additive effects are revealed by planar surfaces, while numerous forms of interactions are revealed by non-linearities in the surfaces. Our use of the response surface approach follows concepts in pharmacology called ‘isoboles’ (Rodea-Palomares et al. 2015) which do not necessarily depend on mechanistic assumptions (IA versus CA) and are independent of the shapes of the dose–response curves so that they might apply for both CA and IA models of additivity (Berenbaum 1981; Rodea-Palomares et al. 2015). Our approach focuses on attention on life history endpoints. When combined with genetic variation and multiple levels of food, we gain further insight into how natural variation in aquatic communities impacts on our assessment of multiple stressors.

## Material and methods

### *Daphnia* culturing

Three genotypes of *D. pulex*, LD33, D86A and D84A, were collected from field populations in the UK and have been in culture for 10 years in the Department of Animal and Plant Sciences, University of Sheffield. These three genotypes have different intrinsic body sizes, life history traits and sensitivity to predation risk (Reger 2013, Reger et al., 2018).

Stock cultures were acclimated in ASTM hard water under controlled conditions of temperature 20 ± 2 °C, photoperiod 16:8 h light:dark and light intensity 140 lux. Prior to experiments, animals were acclimated to test media over 3 weeks as recommended in the OECD guidelines. The cultures were maintained in 2-L tanks with approximately 25 individuals of each *Daphnia* genotypes and fed every day with the green algae *Chlorella vulgaris* fo. *viridis* (strain number: CAAP 211/12). The algae cultures were grown in Ebert medium (Ebert group, Zoologisches Institut Evolutionsbiologie, Switzerland, n.d.) and kept on a table shaker in a controlled room at 20 ± 2 °C under a 16-h light:8-h dark photoperiod with 173 lux.

### Metals preparation

To prepare metals stock solutions, analytical grade dihydrous copper and cadmium chloride (Fisher Scientific, UK) were dissolved in distilled water and these stocks were used to prepare solutions for the experimental treatments. For chronic toxicity exposures (single and mixture), each treatment concentration (single or combined metals) was prepared daily in 1-L volume ASTM hard water and subsequently delivered into experimental jars. Nominal Cu concentrations were 0, 5 and 10 μg/L and nominal Cd concentrations were 0, 0.5 and 1 μg/l. These sub-lethal concentrations were chosen based on an extensive literature review (Sadeq and Beckerman 2019) investigating sub-lethal effects of Cu and Cd on Cladoceran species. All pair-wise combinations of Cu and Cd were assayed, generating a complete response surface grid of nine metal treatments.

### Experimental design

Our exposure tests were performed in accordance with the protocol OECD *Daphnia* sp. No. 212 (OECD, 2012). Our experimental design follows a completely factorial response surface defined by three genotypes, two food conditions (standard food level = 2 × 10^5^ cells/mL and high food level = 5 × 10^5^ cells/ml), and nine metal treatments defined by the three concentrations each of Cu and Cd and their combinations. This standard food level has been used for more than a decade with these genotypes (Beckerman et al. 2010; Dennis et al. 2011; Lind and Jeyasingh 2015; Reger et al. 2018).

We replicated each metal treatment × food level × genotype combination five times for a total of 270 individuals, each observed over 21 days in 100-mL jars (9 metal treatments × 2 food levels × 3 genotypes × 5 replicates). Animals were fed and media were replaced daily.

Actual metal concentrations, estimated from solutions containing algae food resources, were calculated using a separate set of replicate jars using ICP-MS (Inductively coupled plasma mass spectrometry; Agilent 4500; accuracy < 1 ng/L). We estimated realised concentration in media preparations that were prepared exactly as our daily preparations for experiment (see above). Realised concentrations did not deviate from nominal concentrations (linear regression; *n* = 3 per metal concentration; assay 12 hours after media preparation; Cu: *R*^2^ = 0.99, *F* = 1913, *p* < 0.002; Cd: *R*^2^ = 0.98, *F* = 996, *p* < 0.002).

#### Life history traits

We measured five traits in the daphnids over the 21-day experimental period: ingestion rate, size at maturity, age at maturity, somatic growth rate and reproduction as the sum of three clutches. Life history traits in all treatments were assessed by daily observation during the transfer to new media in new jars. Photographs were taken under a microscope Leica MZ6 modular stereomicroscope (GmbH, Wetzler, Germany) with a Cannon EOS 350D DSLR camera. Size at maturity was measured from the top of the head to the base of the carapace spine using ImageJ (Hooper et al. 2006; Beckerman et al. 2010). Age and size at maturity were estimated on the day neonates first appear in the brood pouch. Reproductive output was recorded by counting neonates produced over three clutches. Somatic growth rate was calculated as ln (size at maturity/initial size)/(age at maturity (days)).

Ingestion rate was measured on animals at their day of maturity in all treatments. As media was replaced daily with known food concentrations, this is a straightforward assay recording algae concentration on transfer to the new media and 24 hours later before transfer to new media again. It was calculated as the number of algae cells digested by daphnids over 24 h (cells/h) (final measurement (after 24 h) − initial measurement (0 time)) using spectrophotometry at wavelength 440 nm. (Ferrando and Andreu 1993; Beckerman et al. 2007).

### Statistical analysis

For each trait, we implemented the following analysis pipeline that maps onto our experimental design. We fit a response surface model that combined first order main effects of Cu and Cd, second order polynomials of Cu and Cd, and the interaction between Cu and Cd. This is the classic response surface model used to reveal additive and several forms of interactive (synergistic/antagonistic) effects on the life history endpoints (Khuri and Cornell 1996). We combined this standard response surface model with the main effects of, and interaction between, algae food levels and *daphnia* genotype, algae levels and each of the metals, and *daphnia* genotype and each of the metals. Our statistical model is thus defined as

Trait ~ (Cu + Cu^2^ + Cd + Cd^2^ + Cu * Cd) +

Food Level + Genotype +

(Cu * Food level) + (Cd * Food level) +

(Cu * Genotype) + (Cd * Genotype) +

(Food Level * Genotype)

The terms in the first line define the classic response surface model that allows for planar or non-linear shapes to be detected over the nine treatment combinations for each metal. The second line identifies the additional main effects of food level and genotype and the third–fifth line, the interactions between these terms and between them and the metals. This model allows us to answer the questions a) does the effect of Cu on trait vary by Cd?, b) is the effect of Cu or Cd non-linear?, c) does the effect of Cu or Cd vary by food level?, d) does the effect of Cu or Cd vary by genotype? and e) does the effect of food level vary by genotype?

All data were analysed using the statistical software programme R (R Core team, 2013) version 3.4.2. We fit the model using the lm function (linear model) in R followed by Type II sums of squares implemented by the Anova function in the *car* package for R for significance testing.

## Results

In each of the following sections, we report for each trait on interactions between Cu and Cd, algae levels and genotype, algae levels and the metals, and genotype and the metals for each measured trait. The results for each trait are visualised by a set of six contour plots graphically depicting the shape of the estimated response surface among genotypes and between food levels; each response surface is defined by a set of contours and colours representing fitted/predicted values from the model. Note that in all presentations of the effect of interactions, the ‘:’ is used to represent the interaction.

As expected from previous research and life history theory linked to reduced energy intake, Cu and Cd lowered ingestion, lowered reproduction, delayed maturation, reduced size at maturity and reduced somatic growth rate. These general patterns, however, are underpinned by several interactions among metals and food, metals and genotype, and food and genotype. In the following sections, we detail these interactions and provide associated visualisations of the response surfaces.

### Ingestion rate

**Fig. 1:**
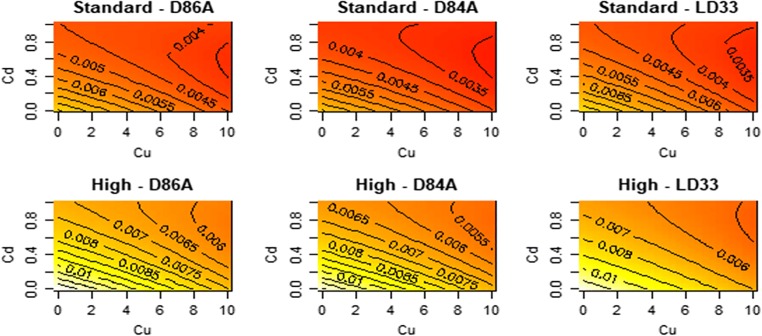
Contour plots for the effect of Cu/Cd mixture on the ingestion rate (cell/h) of different genotypes of *D. pulex*. The plots display the fitted (predicted) values from the response surface model. Each panel is a genotype–food combination and the x- and y-axes are the concentrations of the metals. Yellow versus red colours are higher values of ingestion. Details of significant terms that underpin the shapes that can be seen are described in the text

Overall, ingesting rate decreased with increasing Cu and Cd (Fig. 1). The effects of Cu and Cd on ingestion rates are defined by several interactions (Fig. 1). The two metals interact to shape ingestion rate; the effect of Cu on ingestion varied by Cd in all genotypes (Cu:Cd interaction; *F* = 54, df = 1, *p* < 0.01). Furthermore, the effect of Cd, but not Cu, was non-linear, as indicated by the significant Cd^2^ term (*F* = 10.15, df = 2, *p* < 0.01; t-Cu^2^ = − 0.11, *p* = 0.9, t-Cd^2^ = 4.2, *p* < 0.01). This led to the lowest levels of ingestion occuring at the highest levels of Cu but not Cd.

**Table 1:** The Type II ANOVA table from the response surface model (terms in first column) for each trait.

	Ingestion				Reproduction				Maturation time				Size at maturity				Somatic growth rate			
Term	Sum Sq	Df	*F* value	Pr(> *F*)	Sum Sq	Df	*F* value	Pr(> *F*)	Sum Sq	Df	*F* value	Pr(>F)	Sum Sq	Df	*F* value	Pr(> *F*)	Sum Sq	Df	*F* value	Pr(> *F*)
Cu	0.00001393	1	11.380	< 0.001	32.36	1	50.645	< 0.001	0.09	1	0.248	0.619	0.08632	1	24.169	< 0.001	0.0012639	1	33.071	< 0.001
Cd	0.00008163	1	66.707	< 0.001	45.65	1	71.459	< 0.001	2.26	1	6.183	0.014	0.11271	1	31.559	< 0.001	0.0026278	1	68.761	< 0.001
Cu^2	0.00000002	1	0.014	0.91	6.10	1	9.555	0.002	0.16	1	0.431	0.512	0.00057	1	0.160	0.689	0.0001409	1	3.688	0.056
Cd^2	0.00002152	1	17.589	< 0.001	8.64	1	13.530	< 0.001	0.00	1	0.006	0.938	0.00097	1	0.271	0.603	0.0007434	1	19.453	< 0.001
Food level	0.00042754	1	349.371	< 0.001	13.92	1	21.786	< 0.001	12.87	1	35.223	< 0.001	0.15409	1	43.147	< 0.001	0.0018098	1	47.357	< 0.001
Genotype	0.00000921	2	3.762	0.02	71.27	2	55.777	< 0.001	0.62	2	0.847	0.430	0.04895	2	6.853	< 0.001	0.0001967	2	2.574	0.079
Cu:Cd	0.00006615	1	54.054	< 0.001	124.04	1	194.163	< 0.001	17.58	1	48.101	< 0.001	0.51883	1	145.274	< 0.001	0.008211	1	214.857	< 0.001
Food level:genotype	0.00000388	2	1.584	0.21	0.54	2	0.426	0.654	0.29	20.395	0.674	0.00384	2	0.538	0.585	0.0000069	2	0.091	0.914	
Cu:food level	0.00000622	1	5.083	0.03	0.42	1	0.651	0.421	0.43	1	1.184	0.278	0.03025	1	8.470	0.004	0.0000057	1	0.150	0.699
Cd:food level	0.00001462	1	11.945	< 0.001	0.02	1	0.038	0.845	0.08	1	0.211	0.646	0.031	1	8.679	0.004	0.0000315	1	0.824	0.365
Cu:genotype	0.00000172	2	0.703	0.50	7.26	2	5.682	0.004	1.35	2	1.843	0.161	0.01883	2	2.637	0.074	0.0000062	2	0.081	0.922
Cd:genotype	0.00000055	2	0.226	0.80	5.85	2	4.577	0.011	0.39	2	0.540	0.584	0.00013	2	0.018	0.983	0.0000542	2	0.709	0.494
Residuals	0.00023251	190			120.11	188			69.81	191			0.68213	191			0.0073375	192		

The effect of Cu and Cd also varied by food levels (F-Cu:food level = 5.08, df = 1, *p* = 0.025, F-Cd:food level = 11.9, df = 1, *p* < 0.01). Ingestion declined more with increasing Cu and Cd at high food. In fact, ingestion under metal stress at high foods is reduced to control ingestion rates under low food.

In contrast to the above interactions, the effect of Cu and Cd on ingestion did not vary by genotype (F-Cu:genotype = 0.7, *p* = 0.49; F-Cd:genotype = 0.23, *p* = 0.79). However, the genotypes differ in their average rate of ingestion (*F* = 3.76, *p* = 0.025).

### Reproduction

**Fig. 2:**
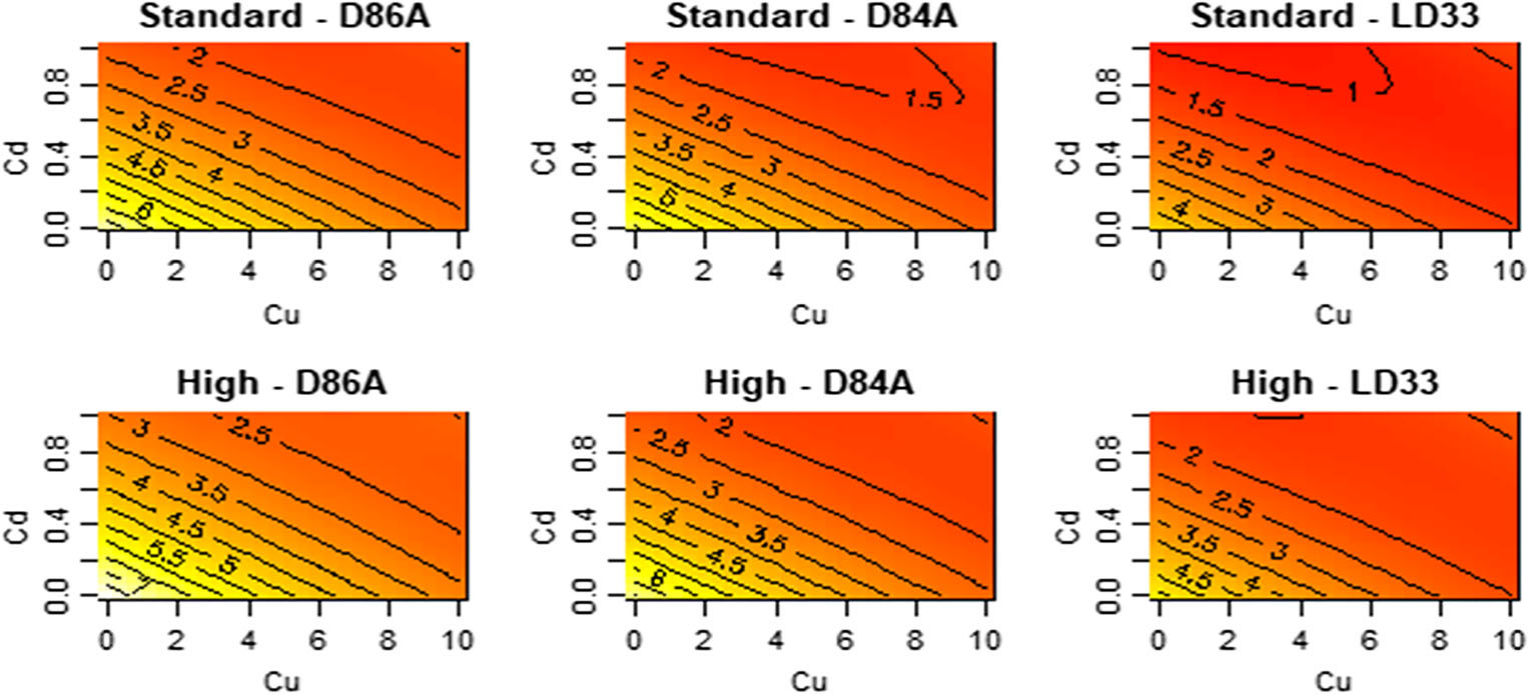
Contour plot for the effect of Cu/Cd mixture on reproduction (mean number of neonates per female) across three genotypes of *D. pulex*. The plots display the fitted (predicted) values from the response surface model. Each panel is a genotype–food combination and the x- and y-axes are the concentrations of the metals. Yellow versus red colours are higher values of reproduction. Details of significant terms that underpin the shapes that can be seen are described in the text

Reproduction declined with increasing Cu and Cd. The effects of Cu and Cd on reproduction are defined by several interactions (Fig. 2). The effect of Cu on reproduction of *D. pulex* varied by Cd; the two metals interact to shape reproduction (Cu:Cd interaction; *F* = 194, df = 2, *p* < 0.01). Further, the effects of Cu and Cd were non-linear, defined by significant squared terms for each (*F* = 18.8, df = 2, *p* < 0.01; t-Cu^2^ = 3.1, *p* = 0.01, t-Cd^2^ = 3.7, *p* < 0.01). Thus, the lowest levels of reproduction occur at the highest levels of Cd, but not Cu.

The effect of Cu and Cd on reproduction varied by genotype (F-Cu:genotype = 5.7, *p* = 0.004; F-Cd:genotype = 4.6, *p* = 0.01). However, The effect of each metal on reproduction did not vary by food levels (F-Cu:food level = 0.65, df = 1, *p* = 0.42, F-Cd:food level = 0.03, df = 1, *p* < 0.84).

### Maturation time

**Fig. 3:**
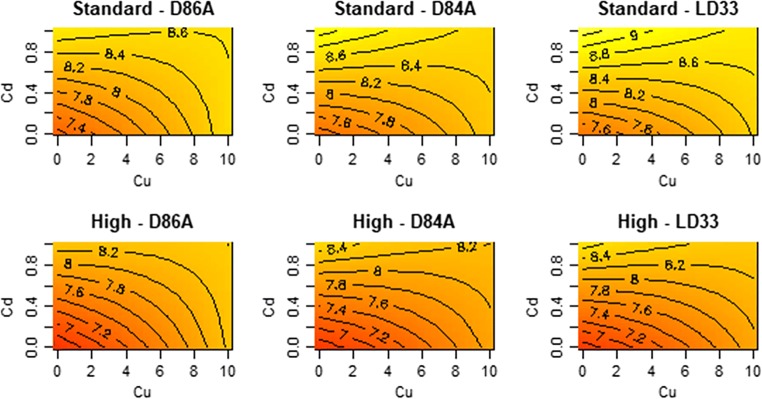
Contour plot for the effect of Cu/Cd mixture on the maturation age (days) of different genotypes of *D. pulex*. The plots display the fitted (predicted) values from the response surface model. Each panel is a genotype–food combination and the x- and y-axes are the concentrations of the metals. Yellow versus red colours are later maturation times. Details of significant terms that underpin the shapes that can be seen are described in the text

Cu and Cd delayed maturation. The effects of Cu and Cd on maturation time are defined by several interactions (Fig. 3). The effect of Cu on maturation time varied by Cd (Cu:Cd interaction; *F* = 84, df = 1, *p* < 0.01). We detected only linear effects of Cu and Cd (*F* = 0.28, df = 2, *p* < 0.76; t-Cu^2^ = 0.66, *p* = 0.51, t-Cd^2^ = 0.07, *p* < 0.94). Figure 3 indicates that maturation was delayed more strongly by Cd than Cu.

Cu and Cd effects did not vary by food levels (F-Cu:food level = 1.81, df = 1, *p* = 0.27, *F*-Cd:food level = 0.21, df = 1 , *p* < 0.64) or by genotypes (F-Cu:genotype = 1.84, *p* = 0.16; *F*-Cd:genotype = 0.54, *p* = 0.58). Maturation was earlier on high food (*F* = 35.22, df = 1, *p* < 0.001). Genotypes did not differ in their maturation time with food levels (*F* = 0.4, *p* = 0.67).

### Size at maturity

**Fig. 4:**
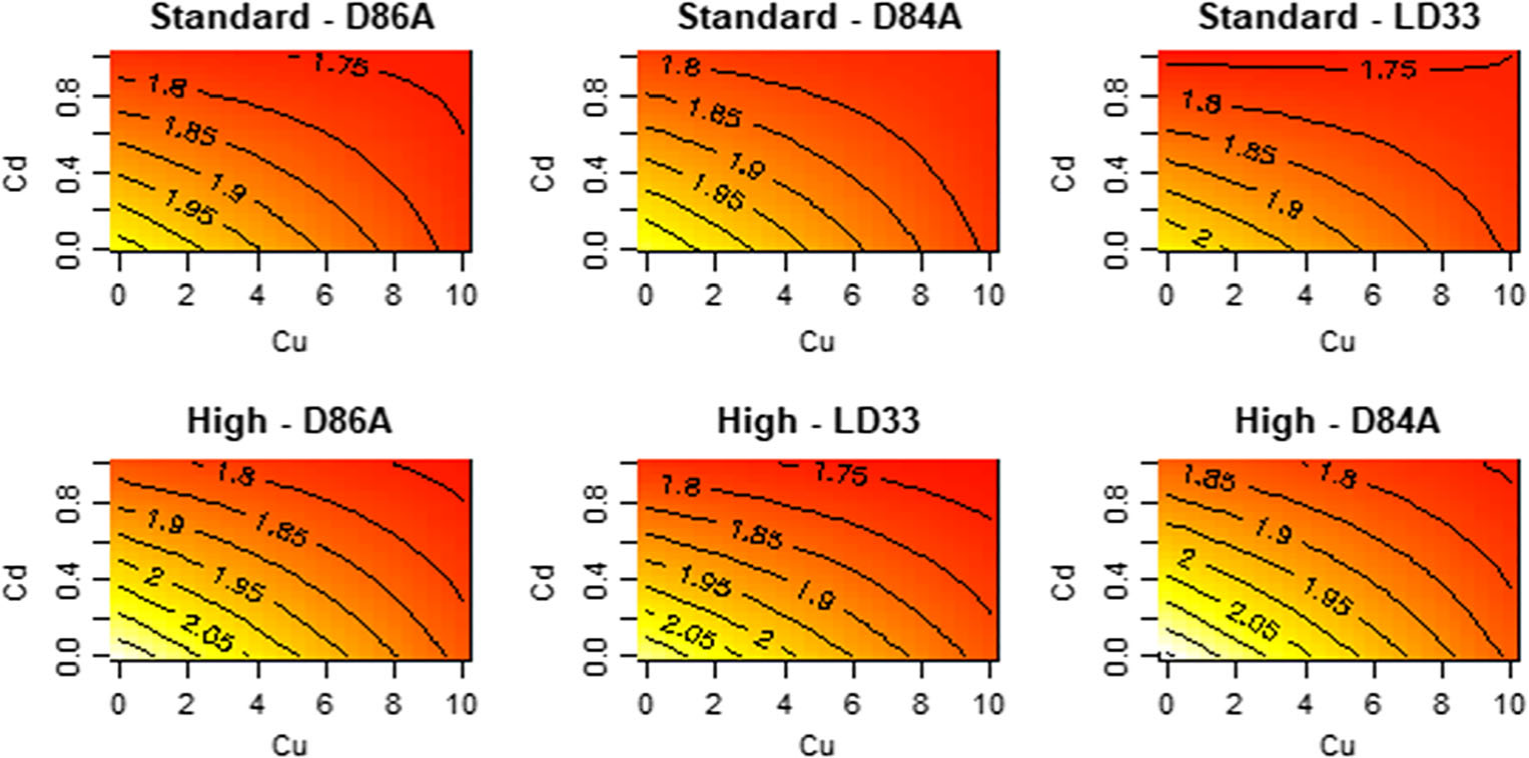
Contour plot for the effect of Cu/Cd mixture on body size at maturity (mm) across three genotypes of *D. pulex.* The plots display the fitted (predicted) values from the response surface model. Each panel is a genotype–food combination and the x- and y-axes are the concentrations of the metals. Yellow versus red colours are larger size at maturity. Details of significant terms that underpin the shapes that can be seen are described in the text

Size at maturity declined with increasing Cu and Cd concentrations. The effects of Cu and Cd on size at maturity are defined by several interactions (Fig. 4). The effect of Cu on size at maturity varied by Cd; size reductions caused by Cd were less pronounced at high levels of Cu (Cu:Cd interaction; *F* = 145.1, df = 1, *p* < 0.01). We detected only linear effects of Cu and Cd (*F* = 0.35, df = 2, *p* < 0.70; t-Cu^2^ = 0.4, *p* = 0.68, t-Cd^2^ = 0.52, *p* < 0.6). The effect of Cu and Cd on size at maturity varied by food level (F-Cu:food level = 8.47, df = 1, *p* = 0.004, F-Cd:food level = 8.68, df = 1, *p* < 0.003). The effect on size at maturity of each metal did not vary by genotype (F-Cu:genotype = 2.64, *p* = 0.07; F-Cd:genotype = 0.01, *p* = 0.98). Average size at maturity did not vary by genotype (*F* = 0.54, *p* = 0.59).

### Somatic growth rate

**Fig. 5:**
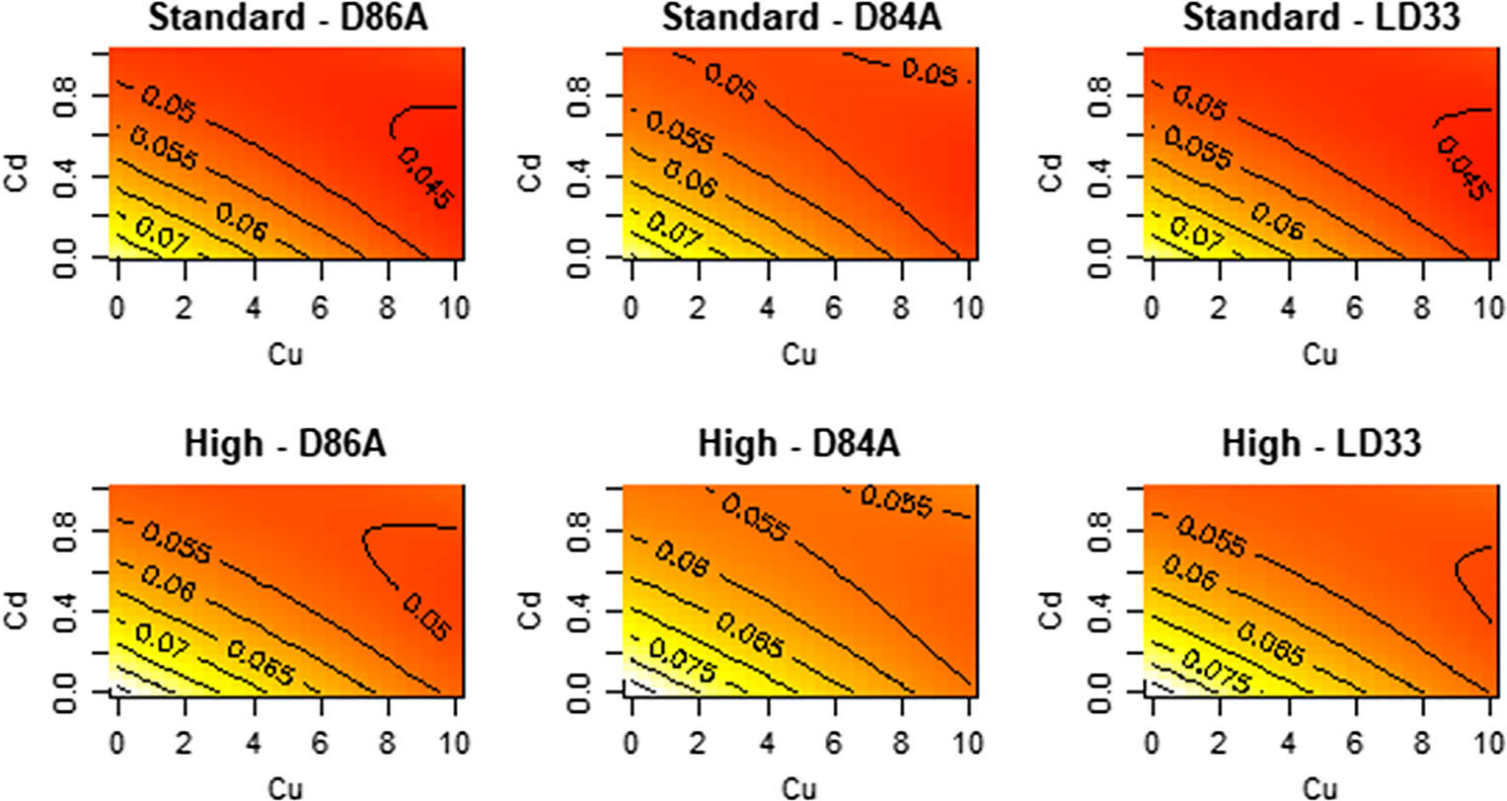
Contour plot for the effect of Cu/Cd mixture on somatic growth rate g (d^-1^) of different genotypes of *D. pulex*. The plots display the fitted (predicted) values from the response surface model. Each panel is a genotype–food combination and the x- and y-axes are the concentrations of the metals. Yellow versus red colours are higher growth rates. Details of significant terms that underpin the shapes that can be seen are described in the text

Growth rates were significantly lower with increasing Cu and Cd. The effects of Cu and Cd on somatic growth rate were defined by several interactions (Fig. 5). The effect of Cu on somatic growth rate varied by Cd (Cu:Cd interaction; *F* = 214.85, df = 1, *p* < 0.01). We detected a non-linear effect of Cd concentration, but not Cu (t-Cu^2^ = 1.92, *p* = 0.56, t-Cd^2^ = 4.41, *p* < 0.01). The effect of Cu and Cd did not vary by food levels (F-Cu:food level = 0.15, df = 1, *p* = 0.7, F-Cd:food level = 0.82, df = 1, *p* < 0.37). Furthermore, the effect of Cu and Cd on somatic growth rate did not vary by genotype (F-Cu:genotype = 0.08, *p* = 0.92; F-Cd:genotype = 0.71, *p* = 0.53). Genotypes did not differ in their average growth rate (*F* = 0.09, *p* = 0.91).

## Discussion

Evaluation of the effect of metal mixtures at sub-lethal levels on life history traits of organisms in aquatic communities is of particular importance to the assessment of ecosystem services (e.g freshwater, recreation), to chemical risk assessment and subsequently, to water quality criteria. In the last few decades, metal pollution has drawn much attention due to their persistence and deleterious effects on aquatic food chains. Though there has been considerable exploration into the toxicity of Cu and Cd on aquatic organisms, particularly Cladocera, the majority of work has focused on single stressors and single genotypes under standardised conditions that are beneficial to a testing environment but not always reflective of natural communities (Brix et al. 2001; Barata et al. 2002; Griffitt et al., 2008; Sadeq and Beckerman 2019). Assessing the interaction among stressors (metals), the role of genetic variation and the impact of variation in food levels remain a key agenda in ecological and ecotoxicological risk assessment.

We assessed the presence of additive versus interactive effects of the metals Cu and Cd on life history of three *D. pulex* genotypes under two food conditions. We employed a response surface experimental design to capture patterns of additive or interactive effects in ingestion rates and life history traits of three *D. pulex* genotypes via a chronic exposure experiment over 21 days. We focused on five classic ecological endpoints linked to daphnid biology and impact in aquatic communities: ingestion rate, reproduction, age at maturity, size at maturity and somatic growth rate.

Despite the presence of several interactions (among stressors, genotypes and food levels), our data indicate (Figs. 1, 2, 3, 4 and 5) that the overall presence of two metals reduced ingestion rates, impaired reproduction, extended the time to maturation, reduced size at maturity and lowered somatic growth rates. The non-linearities we detect do not, we suggest, paint too complex a picture for understanding the combined effects of Cu and Cd. Figures 1, 2, 3, 4 and 5 do not show peaks or troughs of responses at sub-lethal levels of Cu and Cd. There are no saddle points that arise across the concentrations. In general, combined effects of Cu and Cd equate with increases or decreases in the traits we have measured and thus indicate in summary what logic and conventional thinking dictate: a combination of Cu and Cd are substantially worse for *Daphnia* performance.

While, our findings showed a variation in the interaction between metals and food levels and metals and genotypes across traits, our data highlight the importance of context in assessment of life history endpoints in response to different stressors. With respect to Cu, which is known to interfere with digestion, we expected and found that the effect of Cu on ingestion rates varied by food. We also found this pattern of effect for Cd. Metals disrupt digestive physiology, which is linked to energy intake and hence resources acquired for growth and reproductive activities (Barata and Baird 2000; Bui et al. 2016). The relationships between energy intake and the life history traits (more food should increase size at maturity, reduce age at maturity, increase reproduction and increase somatic growth rate) suggests that the interaction between metals and food we see for these traits may arise in part via these relationships between life history and food.

Our work contributes to a growing literature investigating the effects of metal mixtures and *Daphnia* spp. For example, in recent research, binary mixtures of nickel (Ni), zinc (Zn), Cu and Cd produced a more than additive effect on food consumption rate of *Daphnia magna* at low concentration of metals (Lari et al. 2017). Inter-clonal variation in *D. magna* was found in the response of ingestion rate to Cd and temperature (Muyssen and Janssen 2010). This body of data suggests a key role of foraging and digestion in metal effects. Compounding these effects, under metal exposure, organisms may also spend more energy to increase their tolerance (Calow 1991). Supporting our data on reproduction, growth and maturity, data also suggest changes in allocation to growth and reproduction that effect life history responses depending on duration and exposure concentration (Muyssen and Janssen 2001; Bossuyt and Janssen 2004; Canli, 2006; Durou et al. 2005).

Detecting consistency in the type of response (additive, synergistic, antagonistic) is proven challenging. A recent meta-analysis evaluating Cu, Cd and Zn toxicity showed variable interactions for many endpoints measured under the same experimental conditions. The effects were strongly associated with the identity of the endpoint on which the metal combinations were tested (Vijver et al. 2011). Similarly, different patterns of responses have been observed in *D. magna* across traits studied and different metal mixtures (Loureiro et al. 2010; Pavlaki et al. 2011). Data also indicate that interactions between metals may vary from synergistic to antagonistic depending on the metals, properties of contaminants and test species (Shuhaimi-Othman and Pascoe 2007; Meyer et al. 2015; Kim et al. 2017).

While numerous studies found a combination of additive or synergistic effects, many studies have found only antagonistic effects. For example, recent research by Pérez and Hoang (2018) on mixture toxicity to *D. magna* showed that the sub-lethal concentration of Cd and Ni caused antagonistic effects across traits studied. However, other studies on metal mixture toxicity have reported less than additive effects to *D. magna* (Komjarova and Blust 2008; Meyer et al. 2015; Traudt et al. 2016; Pérez and Hoang 2017, 2018). Mahar and Watzin (2005) examined the impacts of Cu, Zn, and insecticide mixtures on the survival and reproduction in *Ceriodaphnia dubia*. It has determined less than additive effect for the binary mixture of Cu and Zn on survival, but more than additive effect on reproduction. Further, an antagonistic effect was observed on reproductive activity of *C. dubia* under exposure to high concentrations of benzalkonium chloride with binary mixtures of anticancer drugs, but additive effects in all mixtures at low concentrations (Russo et al., 2018).

Our research, and that of others noted above, highlights that if we are to move towards generalising and predicting the effect of multiple stressors, experiments need to account not only for the diversity of traits that can be evaluated but also for context defined by the metal identity, concentrations, food levels and genotype.

Interestingly, apart from reproduction, our data show that the effects of metals rarely varied among our three genotypes. While three genotypes is certainly too few to generalise about the nature of genetic variation, the data suggests that we should assess whether uniformity in responses to metals is more common than say, to food or predation. Baird et al. (1990) found small differences among *D. magna* genotypes in response to chronic Cd and 3,4-dichloroanilin. These responses may be related to specific mechanisms of heavy metals (e.g. metallothionein versus detoxification). In contrast, Barata, et al. (2001) suggested that differences in *D. magna* genotypes’ response to ethyl parathion was due to genetic differences in tolerance.

## Conclusion

Our data indicate that the effect of Cu on ingestion rate and life history of *D. pulex* depend on Cd, and that metal effects vary by food levels and genotypes. Despite the pervasive presence of interactions on these traits, the overall picture is that combined metals are worse for the daphnid performance than single metals. We also found that the effects of metals on *D. pulex* varied by food levels in the ingestion rate and size at maturity, but much less so by genotype (reproduction only). This study supports the increasingly called for assessment of multiple performance measures (feeding and life history) under multiple stressors (natural and anthropogenic) and among naturally occurring genotypes from the wild.

## Figures and Tables

**Fig. 6:**
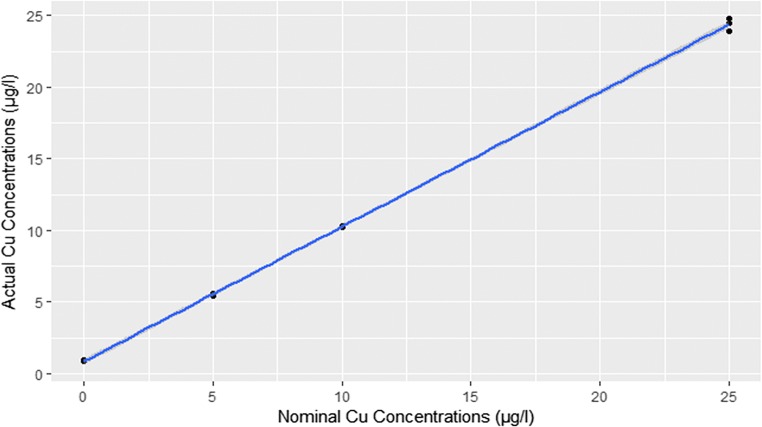
The nominal vs. Actual concentrations for Cu and Cd

**Fig. 7:**
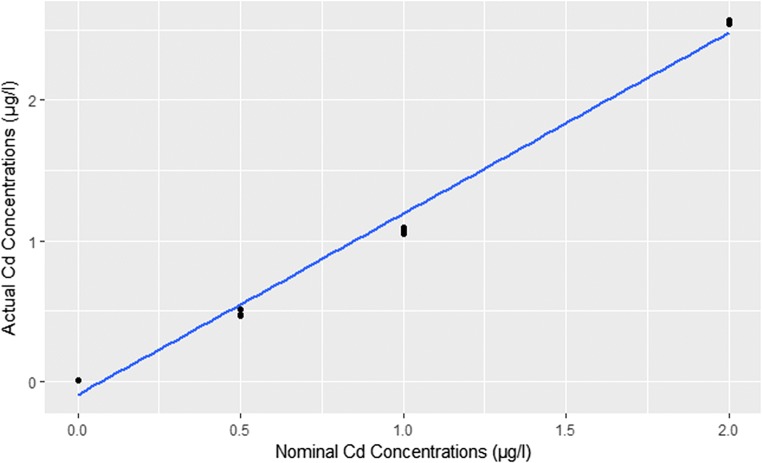
The nominal vs. Actual concentrations for Cu and Cd

## Appendix

### A. Nominal and actual concentrations of copper and cadmium

Throughout the experiments using metals, nominal concentrations of 5, 10 and 25 μg/L of copper(II) chloride dehydrate and concentrations of 0.5, 1 and 2 μg/L of cadmium(II) dehydrate chloride (Fisher Scientific, UK) were delivered in aqueous solution. To prepare metals stock solutions, analytical grade dihydrous Cu and Cd were dissolved in distilled water.

Chronic exposure tests were performed in accordance with the standard protocol OECD Daphnia *sp*., No. 212 (OECD, 2012). Neonates of *D. pulex* (< 24 h), each individual in 100-mL glass jar containing 50 mL of hard ASTM, were exposed to the metals over 21 days with media replaced daily.

We assessed our realised concentrations in the media, in glass jars with algae concentrations at the ‘standard food’ ration (see ‘Material and methods’) using inductively coupled plasma mass spectrometry (ICP-MS). For each metal, realised concentrations were equal to the nominal concentrations. The best-fitting regression line predicts realised concentration = 0.84 + 0.94 * nominal concentration (Fig 6; *R*^2^ = 0.99, *F* = 1913, *p* < 0.002). For Cd, realised concentration = − 0.09 + 1.3 * nominal concentration (Fig 7; *R*^2^ = 0.98, *F* = 996, *p* < 0.002).
